# Surface characterization of curcumin-visualized latent fingerprints on metal surfaces

**DOI:** 10.1007/s00216-026-06445-x

**Published:** 2026-03-22

**Authors:** Kristýna Havelková, Jaroslav Otta, Miroslava Trchová, Tomáš Tobrman, Petr Vrablic, Gabriela Broncová

**Affiliations:** 1 Department of Analytical Chemistry, Faculty of Chemical Engineering, University of Chemistry and Technology, Technicka 5, 166 28, Prague 6, Prague, Czech Republic; 2 Department of Physics and Measurements, Faculty of Chemical Engineering, University of Chemistry and Technology, Technicka 5, 166 28, Prague 6, Prague, Czech Republic; 3 Central Laboratories, University of Chemistry and Technology, Technicka 5, 166 28, Prague, Prague, Czech Republic; 4 Department of Organic Chemistry, Faculty of Chemical Technology, University of Chemistry and Technology, Technicka 5, 166 28,Prague 6, Prague, Czech Republic; 5 Criminalistics Institute Prague, Police of the Czech Republic, Bartolomejska 310/12, 110 00 Prague, Czech Republic

**Keywords:** Curcumin, Forensic science, Latent fingerprint, Metals, Morphology, Surface characterization

## Abstract

Visualization of latent fingerprints (LFPs) on metal surfaces using curcumin (CUR) remains a largely underexplored approach. Here, CUR was applied for LFP visualization on brass, steel, Al, and Cu plates and on real-life metal objects (keys, coins, and knives). Fingerprint quality was assessed by stereomicroscopy and characterized in detail using profilometry, SEM/EDS, and vibrational spectroscopy (IR and Raman). Profilometry and SEM/EDS revealed substrate-dependent differences in CUR layer morphology, roughness, and aggregation, indicating that papillary ridge contrast is governed by interactions between fingerprint residues and the underlying surface. Vibrational spectroscopy confirmed the preferential interaction of CUR with lipid-rich LFP components rather than non-specific adsorption on metal substrates and revealed compositional differences in fingerprints deposited on brass and stainless steel. The practical applicability of CUR on real-life metal objects demonstrated that fingerprint persistence and visibility met the legal requirement for forensic usability in the Czech Republic (> 10 minutiae). Overall, these results demonstrate that, under appropriate surface conditions, CUR provides a reliable and environmentally sustainable strategy for targeted LFP visualization on metal substrates.

**Graphical abstract:**

**Figure:**
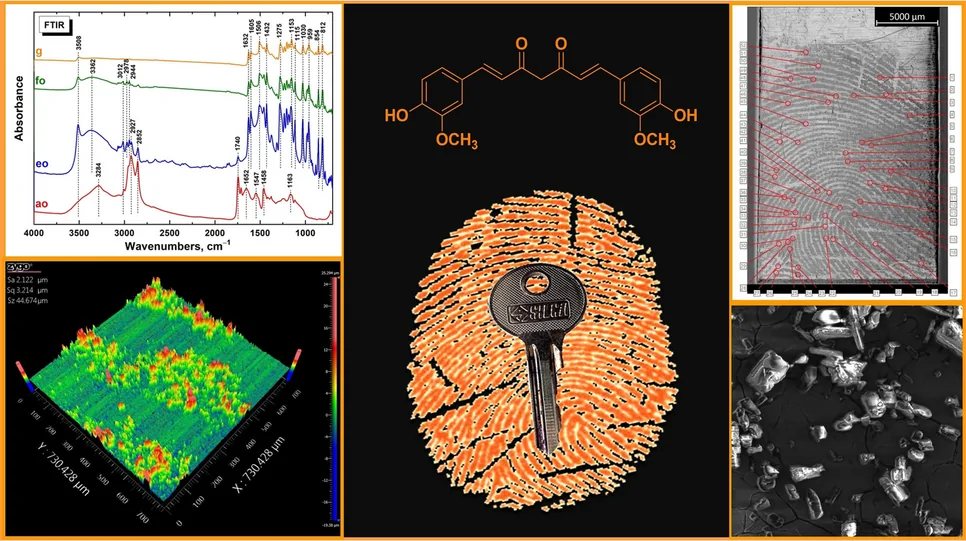

**Supplementary Information:**

The online version contains supplementary material available at https://doi.org/10.1007/s00216-026-06445-x.

## Introduction

**Fig. 1:**
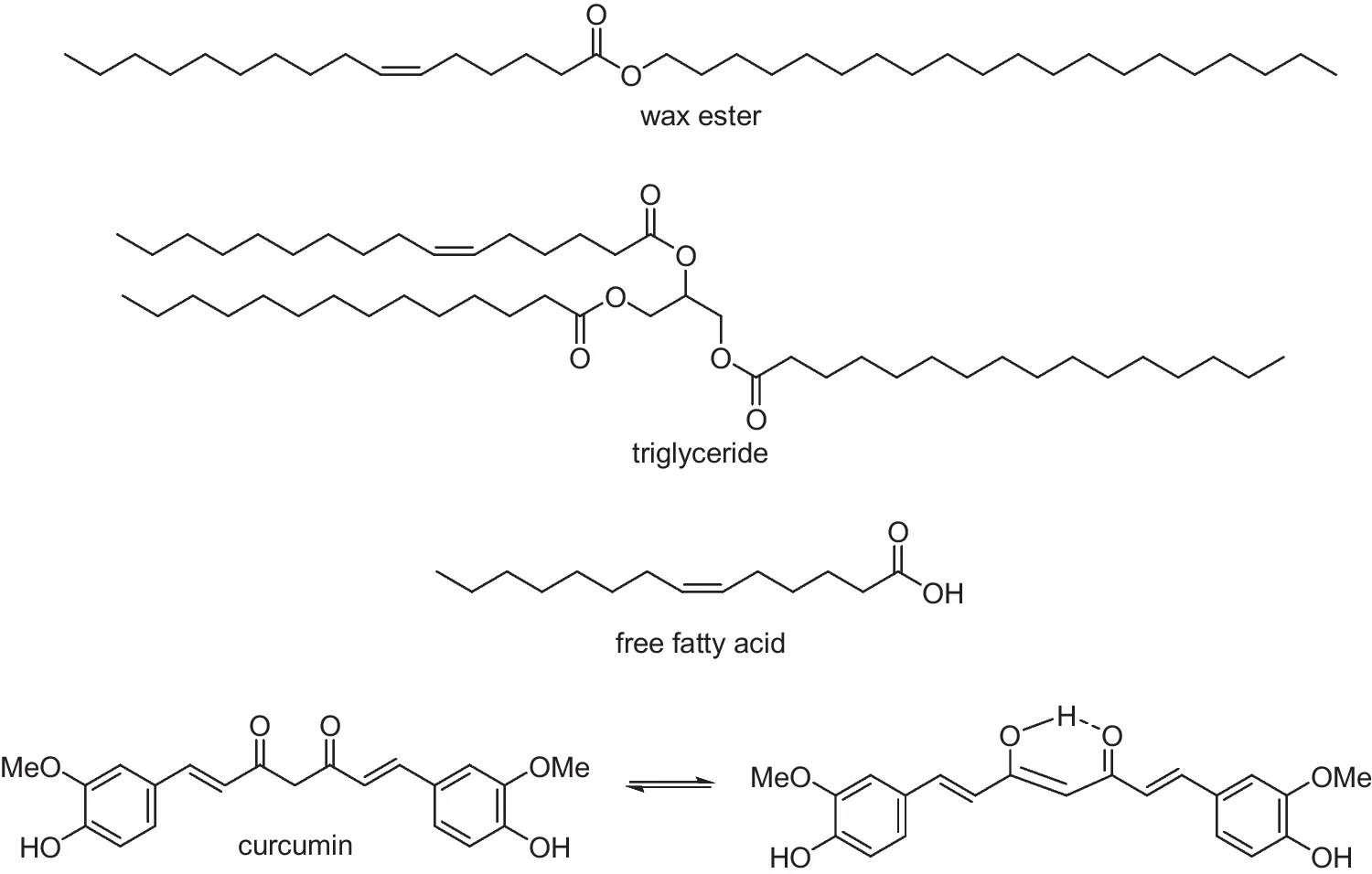
Chemical structure of curcumin and representative components of lipophilic fraction

Despite the rapid development of DNA profiling, latent fingerprints (LFPs) remain among the most frequently used identification traces in forensic analysis. Visualization of LFPs at sufficient quality is crucial for subsequent dactyloscopic identification and interpretation. The choice of method for LFP visualization is closely related to fingerprint composition. The key components of fingerprints are eccrine sweat and skin debris. Eccrine sweat primarily contains inorganic ions, amino acids, urea, lactic acid, and creatine [1]. In contrast, the lipophilic fraction is mainly composed of wax esters, triglycerides, free fatty acids, squalene, and cholesterol esters (Fig. 1) [2]. A wide range of techniques is currently used in practice to detect LFPs, including powder methods [3], cyanoacrylate (CA) fuming [4], and dyes [5]. Although these procedures are well established, many rely on organic solvents, synthetic dyes, or other chemicals that may be toxic and pose environmental burdens [6].

Accordingly, incorporating green chemistry principles into forensic practice represents an important and timely direction. In the context of LFP visualization, this involves identifying non-toxic, biodegradable, and highly effective agents that provide imaging quality comparable to or better than traditional methods [7]. Special attention is therefore given to natural dyes and materials whose chemical structures allow interaction with lipids, amino acids, and other fingerprint components, providing sufficient contrast against the substrate [8, 9].

Recent studies have demonstrated the forensic potential of plant-derived materials. For example, Sekar et al. showed that finely ground durian seeds are effective on both porous and non-porous surfaces [10]. King et al. reported that fluorescent pigments in Spirulina exhibit strong near-infrared fluorescence and enable high-quality visualization of LFPs on porous and semi-porous substrates [9]. Passos et al. compared several natural algal powders on glass and confirmed the superior contrast of *Spirulina* sp. over *Chlorella* sp. and *Laurencia dendroidea* [11]. Pigments from purple sweet potatoes were also found to be particularly suitable for non-porous materials [12]. This trend toward sustainable colorants has extended to green nanotechnology approaches [13]. Elipe et al. demonstrated that nanoparticles derived from plant waste (rice husks or neem leaves) can achieve up to 90% readability of LFPs on various surfaces, reducing the toxicity of conventional visualization agents [14]. Common plant spices and leaves have also attracted interest. Nicolodi et al. demonstrated the effectiveness of powders from *Curcuma longa* (turmeric), *Murraya koenigii* (curry), *Cinnamomum verum* (cinnamon), *Laurus nobilis* (bay leaf), and *Capsicum annuum* (pepper) in detecting LFPs on plastics and glass [8].

Curcumin (CUR), the main polyphenolic pigment of *Curcuma longa*, has emerged as a highly promising agent for LFP visualization [15]. CUR exhibits pronounced optical properties, intense yellow–orange color, strong fluorescence, the ability to form complexes with metal ions, and a lipophilic nature [16]. These characteristics make it a potential candidate not only for pharmaceutical and medical applications [17], but also in forensic science, where it meets the criteria of an environmentally friendly dye [18].

Structurally, CUR contains a β-diketone group and two phenolic units, which can interact preferentially with lipid and protein components of LFPs [19]. Such interactions facilitate selective staining of papillary ridges. Previous studies have demonstrated that CUR and its derivatives can effectively visualize LFPs on a wide range of non-porous and porous substrates, including glass, plastic, and Al [20, 21]. Additionally, Durgi et al. synthesized two CUR analogs with enhanced emission properties and showed that these compounds function as highly efficient fluorescent probes, improving the visibility of ridge details under UV irradiation and expanding the potential of fluorescent detection for LFPs [22]. Further developments have focused on composite materials to enhance imaging performance. For instance, Prabakaran et al. described a CUR-based nanocomposite system for improved LFP detection [23], while Wang et al. introduced a CUR/kaolin composite that offered advanced fluorescence quantification and overall superior visualization compared to conventional developing agents [24].

Despite these advances, comparatively little attention has been paid to CUR’s application on metallic surfaces. Visualization of LFPs on metallic substrates remains challenging due to the strong influence of the physicochemical properties of metals, such as electrical conductivity, smooth surface morphology, and chemical reactivity on fingerprint adhesion, stability, and overall readability [25]. Published studies on CUR have primarily focused on powder-based methods [18], which are highly effective on various substrates but may face practical limitations on metals, including particle adhesion, uniform coverage, and potential mechanical disruption of fragile FPs [26]. Limitations in the visualization of LFPs on metal surfaces represent a significant challenge in forensic science, as numerous metal objects—including weapons, knives, cartridge cases, and other metallic tools—are commonly encountered at crime scenes.

**Fig. 2:**
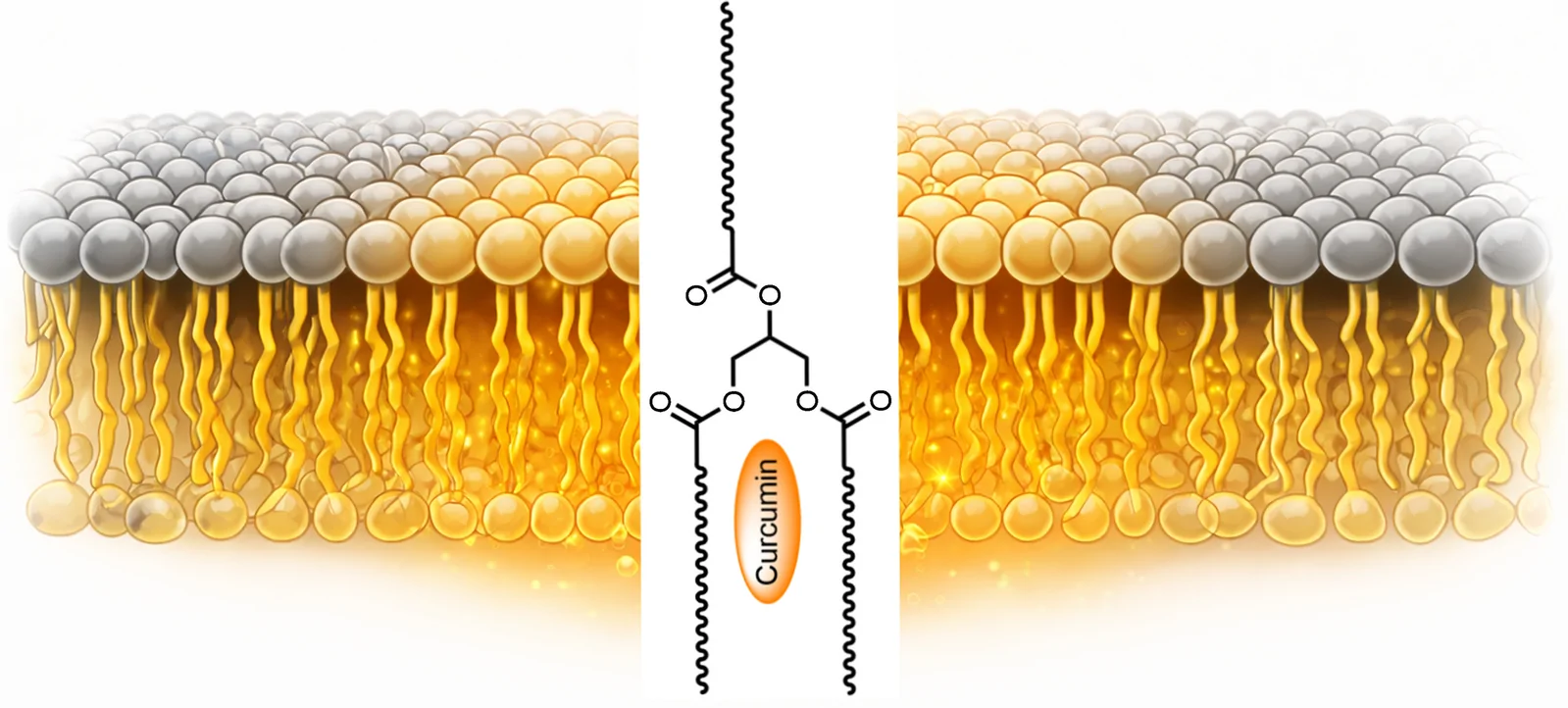
Schematic representation of a curcumin–lipid non-covalent complex

Despite growing interest in using CUR for LFP visualization, a comprehensive characterization of LFPs developed with CUR solution on metal substrates is still lacking. In this context, it should be noted that CUR represents a promising reagent for the visualization of LFPs on metal surfaces because of its preferential interactions with lipid components of fingerprint residue via hydrophobic interactions and hydrogen bonding [27]. These non-covalent interactions enable CUR to bind to the lipid layer with a binding constant of 4.26 ± 0.12 × 10^4^ M⁻^1^ [28]. Although the exact binding site of CUR in lipid systems depends on the alkyl chain length, experimental studies have demonstrated that CUR predominantly associates with the hydrophobic region of the alkyl chain in close proximity to the polar glycerol group (Fig. 2) [28]. Here, we present a systematic study combining SEM, EDS, profilometry, and vibrational spectroscopy (IR and Raman) to provide a detailed characterization of LFPs visualized with CUR on metal surfaces. These findings contribute to an objective assessment of the potential of this sustainable and effective forensic method, which combines analytical performance with environmental responsibility.

## Experimental section

### Chemicals and substrates

Curcumin (CUR) was sourced from Sigma-Aldrich (China). Acetone, used for substrate cleaning, was supplied by Lach-Ner (Czech Republic (CZ)). The solutions were prepared using redistilled water (conductivity, 2.3 µS·cm⁻^1^ at 22 °C) and 96% p.a. ethanol (Lach-Ner, CZ).

To optimize LFP visualization conditions, four metallic materials commonly found in everyday life were selected: brass, steel, Cu, and Al (Woodgrain s.r.o., CZ). These 0.5-mm-thick materials were cut into plates measuring approximately 15 × 25 mm.

### Preparation of substrates and LFP deposition

All metal plates were cleaned sequentially by immersion in soapy water, acetone, redistilled water, and ethanol, and allowed to dry at laboratory temperature before fingerprinting. LFPs were applied by four donors (females, 20–45 years), one of whom was wearing cosmetic makeup (L’Oréal Paris, True Match). Before application, hands were washed with soap and air-dried. The fingers were then rubbed on the face to obtain natural skin residues, followed by transferring the LFPs to the cleaned metal substrates. The LFP deposition procedure followed commonly used protocols for natural sebaceous LFPs described in the forensic literature and recommended by the International Fingerprint Research Group (IFRG) [29, 30].

A systematic aging study of sebaceous LFPs visualized by chemical deposition of CUR on metallic substrates was performed. LFPs were deposited on metal plates according to the procedure described above and stored under laboratory conditions. The LFPs were aged for 1, 3, 7, and 14 days prior to visualization. After the aging periods, the LFPs were visualized using the CUR deposition procedure. The developed LFPs were evaluated visually using a stereomicroscope. Changes associated with aging were further quantified using profilometry; each substrate was measured six times to ensure reproducibility.

The chemical composition of the substrates and real objects was verified using X-ray fluorescence (XRF). Detailed elemental composition data and the corresponding spectra are included in Table S1 and Fig. S1 (see Supplementary Materials).

To verify the method’s applicability, the same preparation procedure was applied to real metal objects (key, dining knife, Czech coins) composed of metals of similar composition.

### Visualization procedure

CUR solutions were prepared at concentrations of 8.1 mM and 16.3 mM by dissolving the dye in ethanol/water mixtures. Several ethanol/water volume ratios were tested, ranging from 1:2 to 2:1. The selected concentrations were established based on preliminary optimization experiments aimed at maximizing ridge-to-background contrast while minimizing non-specific background staining. Lower concentrations resulted in insufficient ridge development, whereas concentrations above 16.3 mM did not provide further improvement in contrast and occasionally increased background coloration. The chosen range is also consistent with concentration levels reported for lipophilic dyes applied in fingermark visualization [1, 31].

Model metal plates bearing LFPs were placed in glass pill bottles, whereas real metal objects were placed in Petri dishes or test tubes (Fig. S2, see Supplementary Materials), depending on their size. Subsequently, 5–6 mL of CUR solution was added to each vessel, with the volume adjusted according to the size of the object and the solution concentration. The metal substrates were immersed in the dye solution and periodically inspected every 10 min, with a total exposure time ranging from 10 to 60 min. After removal at appropriate time points, the substrates were rinsed with redistilled water in a beaker and allowed to dry in air at laboratory temperature before further analysis. Accordingly, the visualization procedure was based on previously reported dye-immersion methods for latent fingermark development on non-porous substrates [32].

### Morphological and structural characterization

#### Profilometry

The analysis of the topography of visualized LFPs on metallic model substrates was performed using the NewView 9000 optical profiler from Zygo (USA) and the white-light interferometry method. The instrument is equipped with optics offering 10× magnification and a numerical aperture of 0.28, enabling three-dimensional surface imaging with a vertical resolution of up to 0.1 nm. The size of the acquired images was 1850 × 1850 µm. For the quantitative evaluation of the roughness parameters such as Sa (arithmetical mean height) and Sq (root mean square height), as well as spatial frequencies, the Zygo Mx software with the microstructure metrology module was used.

#### SEM

The enhancement of LFPs using dye deposition was investigated through SEM and energy-dispersive X-ray spectroscopy (EDS). SEM imaging was conducted with a Tescan MIRA 3 electron microscope (FE-SEM, Tescan, Brno, CZ), equipped with both an in-beam secondary electron detector and an Everhart–Thornley secondary electron detector. Magnifications of up to 50,000 × and accelerating voltages of 5 kV (Schottky emitter) were employed to visualize the surface topography of the dye-enhanced LFPs.

#### EDS

The visualized LFPs were deposited on various substrates, such as Al, brass, steel, and Cu. Chemical characterization of the dye-enhanced LFPs was carried out using the Quantax 200 energy-dispersive X-ray spectrometer (Bruker Nano GmbH, Berlin, Germany) equipped with a Bruker XFlash 6 detector. Elemental mapping and chemical analyses were performed at accelerating voltages of 5 kV and 15 kV, with an interaction depth of 0.2–1.9 µm and an interaction radius of 0.09–1.1 µm.

#### FTIR

The spectra of the layers deposited on brass and steel supports were obtained with an FT-IR microscope Nicolet iN10 MX (Thermo-Nicolet, USA) using an MCT-A liquid-nitrogen-cooled detector in the 4000–650 cm^–1^ region with maximum spectral resolution of 0.4 cm^–1^. A reflection technique with a spatial resolution of 25 µm was used. The spectra were corrected for CO_2_ and humidity in the optical path.

#### ATR FTIR

The spectra of the powder CUR and layers deposited on brass and steel substrates were analyzed using a Nicolet 6700 spectrometer (Thermo-Nicolet, USA) equipped with a reflective ATR extension GladiATR (PIKE Technologies, USA) with a diamond crystal. Spectra were recorded in the 4000–400 cm^–1^ region with DLaTGS at a resolution of 4 cm^–1^, 64 scans, and Happ-Genzel apodization. Contributions of humidity and CO_2_ were eliminated. One part of the spectra from 2400 to 2100 cm^–1^ was removed due to an ATR crystal artifact.

#### Raman

The spectra were collected using a Thermo Scientific DXR Raman microscope equipped with a 780 nm laser excitation. The laser power at the sample surface was 5.0 mW. The laser beam was focused with a 50× objective of an Olympus confocal microscope, and Raman scattering was detected with a multichannel thermoelectrically cooled CCD camera. The scattered light was analyzed using a spectrograph with a holographic grating with 400 lines per mm and a pinhole width of 50 µm. The acquisition time was 10 s with 10 accumulations.

#### Dactyloscopic comparison

The assignment of numbers to individual minutiae was performed using the digital fingerprint comparison device DactyScope Pro and the LUCIA Forensic program. The Lucia software was used for rapid, efficient real-time comparison of fingerprint images from any source. This device and software are available at the Institute of Criminalistics of the Czech Police.

## Results and discussion

### Visualization of LFPs on metal substrates

The applicability of CUR for LFP visualization on metallic substrates was evaluated using both model metal plates and real-life metal objects, enabling the assessment of substrate-dependent performance under controlled and realistic surface conditions.

Optimal LFP visualization performance was achieved using a curcumin concentration of 16.3 mM in an ethanol–water mixture with a volume ratio of 2:1 (ethanol:H_2_O). This mixture provided sufficient solubility of CUR, effective interaction with lipid-rich LFP residues, and acceptable background levels and was therefore used for all subsequent experiments. Based on previous studies, Bleay et al. [25] reported that wetting times as short as 10 s are sufficient for porous surfaces, whereas longer immersion times may be required for non-porous substrates. In line with this, Yong et al. [33] employed a 10-min immersion for metallic surfaces. Therefore, this duration was selected as the minimum deposition time in this study. The optimized deposition times of CUR deposition on LFP for both model substrates and real objects are summarized in Table S2 (see Supplementary Materials).

**Fig. 3:**
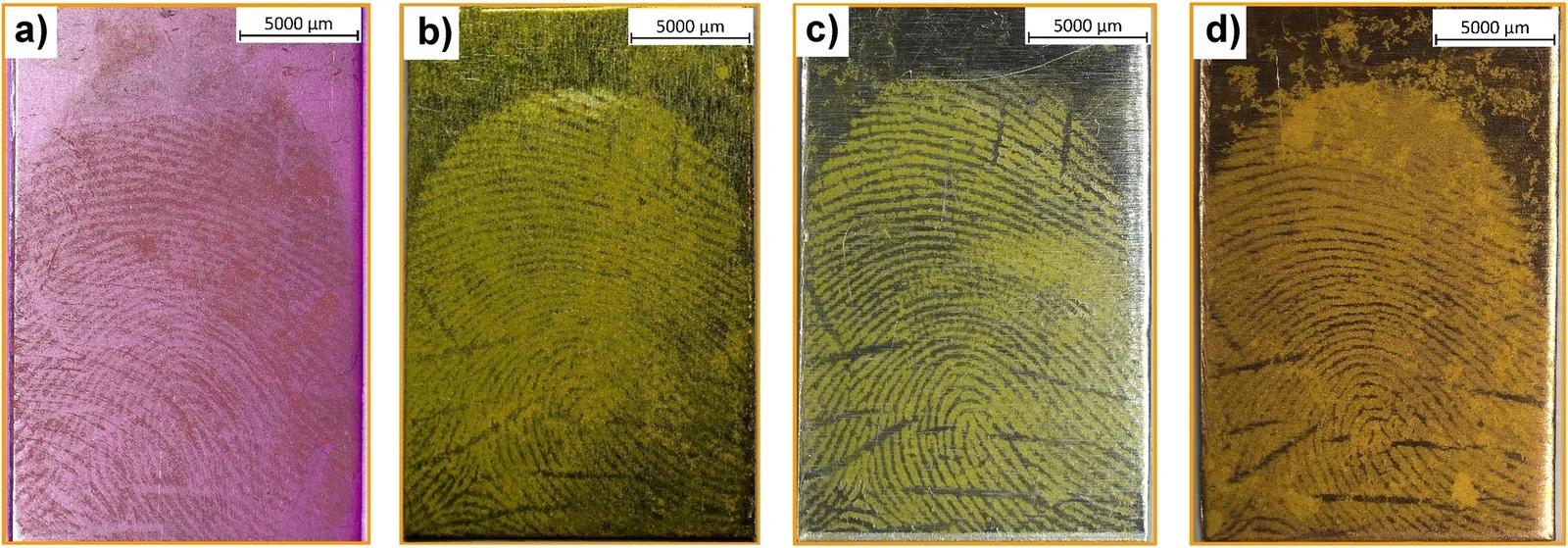
Developed sebum-rich LFPs on **a** steel, **b** brass, **c** aluminum, and **d** copper using CUR. Enhanced contrast on steel under UV illumination

On stainless steel model substrates (Fig. 3), papillary ridge contrast under visible light was limited but significantly enhanced under UV illumination due to the intrinsic fluorescence of CUR. In contrast, UV illumination was not required for Al, Cu, or brass substrates, as these surfaces provided sufficient ridge-to-background contrast under visible light alone. Aluminum substrates (Fig. 3) consistently provided the highest ridge-to-background contrast, indicating a strong preferential interaction between CUR and lipid-rich LFP residues. Notably, despite the similar coloration of the metal substrates and the yellow CUR layer, high-quality visualization of LFPs with well-defined papillary ridges was achieved on both Cu and brass surfaces (Fig. 3).

**Fig. 4:**
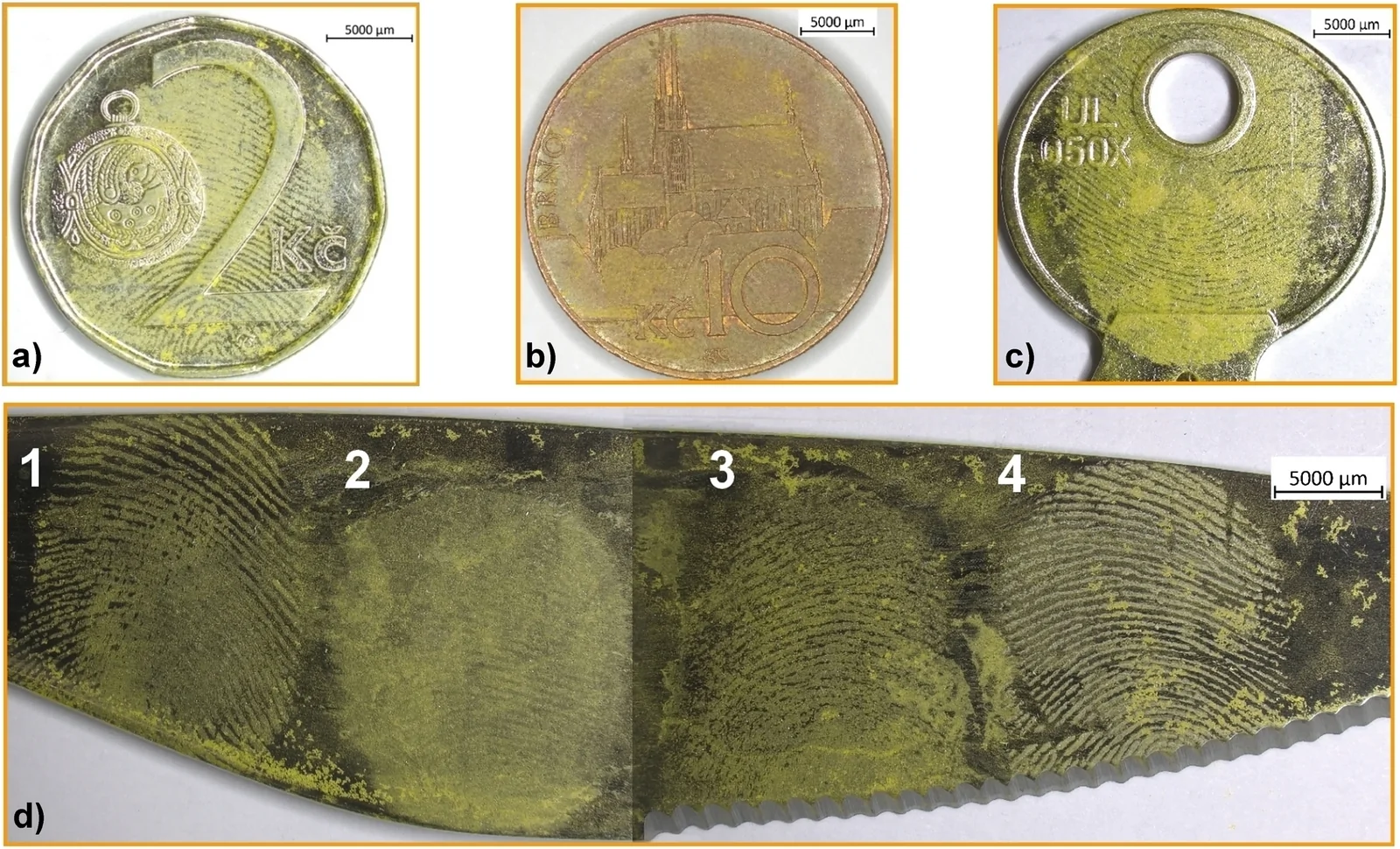
Developed LFPs using CUR on **a** 2 CZK coin, **b** 10 CZK coin, **c** a key, and **d** a dining knife

In contrast, CUR did not provide selective visualization on circulated Cu and brass coins (Fig. 4), where extensive non-selective background staining obscured ridge detail. This behavior differed markedly from that observed on circulated Ni-plated coins (Fig. 4), as well as on stainless steel keys, where high-quality ridge detail was preserved. Although high-quality visualization was achieved on intact Cu and brass model plates, the reduced performance of CUR on Cu- and brass-based coins is likely governed by differences in surface chemistry. Cu- and Zn-containing alloys are prone to oxidation and corrosion and may interact with CUR via metal–ligand complexation, leading to competitive adsorption and loss of selectivity toward LFP residues. Ni-plated and stainless steel surfaces appear to limit such interactions, enabling preferential association of CUR with organic LFP components even on circulated objects.

The influence of LFP residue composition was further examined on stainless steel knives (Fig. 4). Sebaceous-dominated LFPs yielded high-contrast ridge patterns, whereas LFP contaminated with cosmetic makeup (Fig. 4d2) showed reduced clarity, likely due to partial solubilization and redistribution of cosmetic components during the visualization process.

Compared to established techniques, such as CA fuming [4] and small particle reagent (SPR) [34], the CUR visualization process exhibits more pronounced substrate-dependent limitations. While CA fuming provides robust visualization across a wide range of metallic surfaces via polymer film formation, SPR is generally less affected by surface chemistry, but both methods involve either permanent surface modification or reduced selectivity toward lipid-rich residues. CUR offers a single-step, fluorescence-based alternative aligned with green chemistry principles, causing minimal surface modification. Additional references describing standard methodologies for LFP enhancement have been included, and a comparative table summarizing the performance of CUR versus other developer agents on different metallic substrates is provided in Table 1.

**Table 1:** Summary of selected methods and substrates tested for LFP development on metals

**Material**	**Brass**	**Steel**	**Cu**	**Al**	**Aging**	**Ref**
**Method**						
Chemical deposition of CUR	This work	This work	This work	This work	1–14 days	This work
Turmeric powder	-	Painted steel	-	Yes	Not reported	[18]
Cyanoacrylate fuming/Basic Yellow 40	Yes	Yes	-	-	1–14 days	[35]
Gentian Violet	-	-	-	Yes	1 day–12 months	[36]
Sudan Black Oil Red O Gentian Violet	-	Yes	-	-	1 day–2 months (burial period)	[33]

The LFPs visualized using CUR were subsequently subjected to further morphological and chemical characterization, which is presented in the following section.

### Morphological and structural characterization

#### Profilometric characterization

**Fig. 5:**
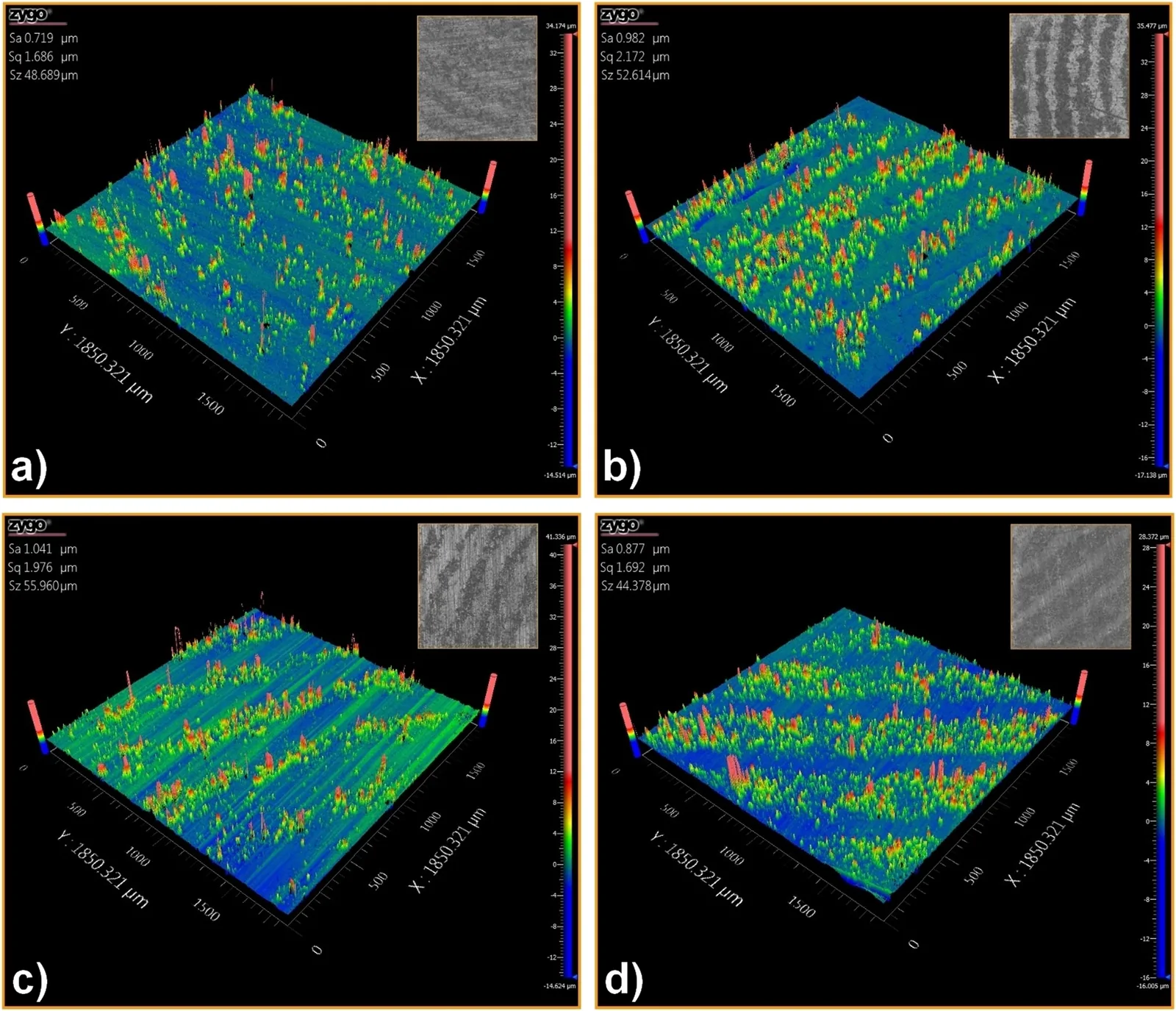
Profilometric images of the interface between the metal substrate and the LFPs developed using CUR: **a** steel, **b** brass, **c** aluminum, and **d** copper

The profilometric analysis of the metal plate surfaces with visualized LFPs exhibited discrepancies in the sorption behavior of the CUR across diverse substrates. As illustrated in Fig. 5, the measurements consistently encompassed the interface between the substrate surface and the fingerprint area.

The CUR dye demonstrated a maximum profile height ranging from 44.0 to 56.0 µm across all tested materials. This observation aligns with findings in other studies where roughness and surface topography are recognized as influential factors for adhesion and interaction of materials with biological properties, such as amorphous carbon films, functionalized Ti surfaces, or polydimethylsiloxane (PDMS) [37]. As such, the presence of micro-irregularities can foster enhanced dye adhesion, given that larger surface areas allow for more significant interaction with the dye particles [38]. Figure 5 shows that CUR formed smaller clusters of particles on the steel surface.

This may be the reason for the lower contrast of the LFPs. Comparatively, on brass, Al, and Cu substrates, the distribution of CUR particles was significantly higher, leading to a more uniform dye layer and distinctive visibility of papillary lines. The findings suggest that surface roughness variance affects not only dye adhesion but also its visual outcome, as evidenced by the defined characteristics of the papillary patterns.

#### SEM characterization

**Fig. 6:**
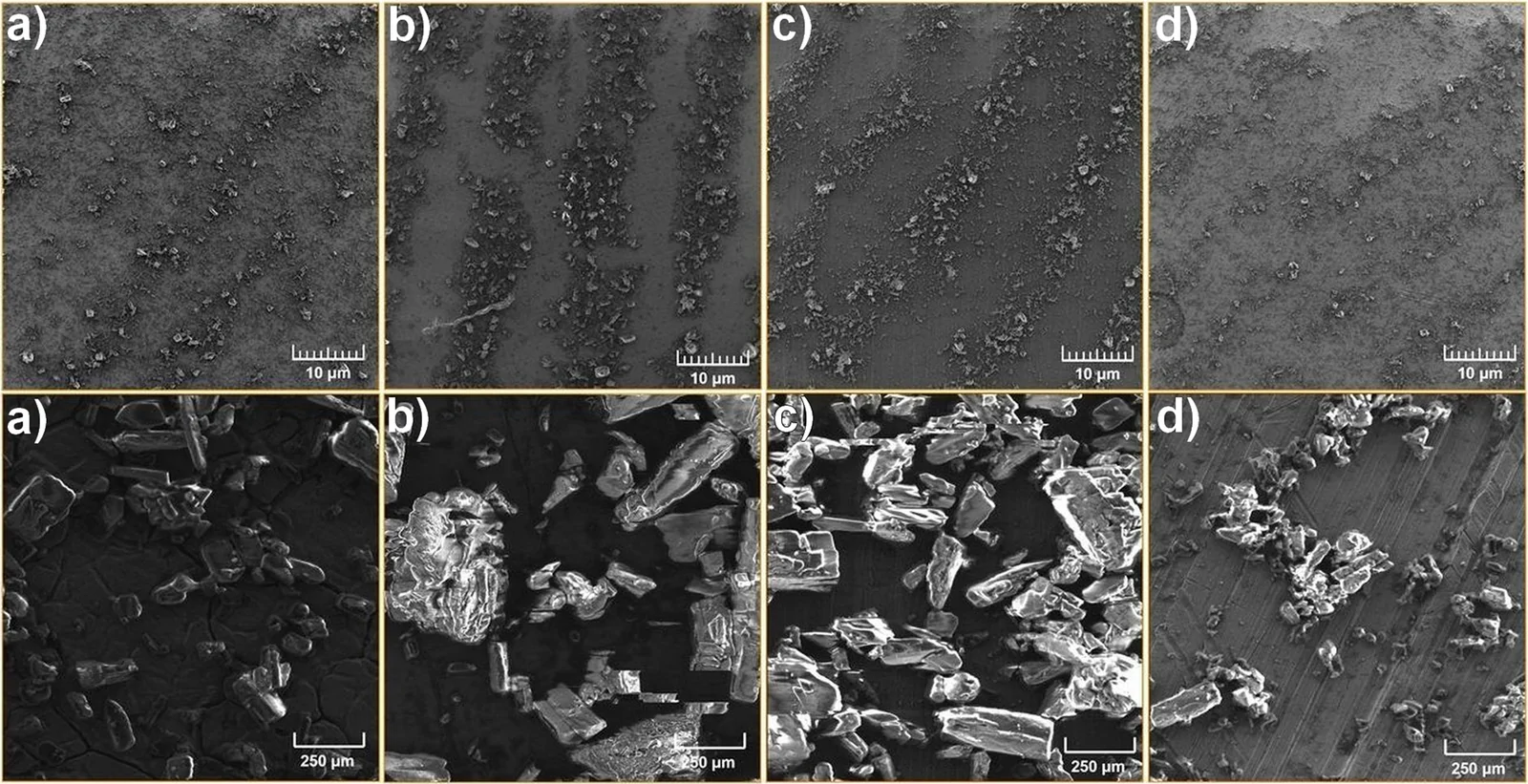
SEM images of the interface between the metal substrate and the LFPs developed using CUR: **a** steel, **b** brass, **c** aluminum, and **d** copper

SEM images obtained at 200 × and 5000 × magnifications were utilized to analyze the surface morphology of the visualized LFPs and metal substrates (Fig. 6). The 200 × magnification facilitated the identification of the overall distribution of the dye and the presence of the fingerprint, while the 5000 × images, when examined in detail, reveal subtle structural differences, morphology, and the way the dye adheres to the surface and fingerprint.

CUR, a notable polyphenolic compound, is characterized by its strong tendency to crystallize, as illustrated by the SEM images (Fig. 6) which depict well-defined crystals. These crystals exhibit sharp edges and irregular shapes. This aligns with the known crystallization behavior of CUR under conditions of surface-induced heterogeneous nucleation, where the substrate interface plays a decisive role in initiating and directing crystal growth, rather than spontaneous bulk nucleation in solution [39]. The most pronounced crystal structures were observed on visualized LFPs on brass, Cu, and Al, whereas steel exhibits a much finer and more homogeneous crystal layer. These differences reflect substrate-dependent interactions governed by surface energy, microstructure, and reactivity. Non-ferrous metals appear to promote larger CUR aggregates, consistent with their favorable surface chemistry and propensity for enhanced structuring during adsorption or initial corrosion-related processes.

In contrast, the finer CUR distribution on steel likely arises from its higher mechanical robustness, distinct microtexture, and comparatively weaker affinity for foreign organic residues in fingerprint material. These surface characteristics create a more uniform adsorption environment, suppressing the formation of large crystalline domains. Overall, the contrasting crystal morphologies highlight how metal composition and surface properties fundamentally shape CUR nucleation and growth on fingerprint residues.

In addition to the dye distribution, the steel substrate exhibits more pronounced surface heterogeneity in the SEM images (Fig. 6) compared to brass, Al, and Cu. As no chemical or electrochemical etching was performed prior to imaging, these features cannot be attributed to metallographically revealed grain boundaries. Instead, they most likely reflect differences in surface finish, manufacturing treatment, and possible oxide-related heterogeneity of the commercially supplied steel sheets. Such surface irregularities may influence the spreading and anchoring of fingerprint residues, leading to locally variable CUR deposition. In contrast, brass, Al, and Cu display a comparatively more homogeneous surface appearance under the same imaging conditions, where predominantly overall surface roughness is observed. The more uniform surface characteristics of these substrates may facilitate more consistent deformation marks and dye distribution, which is reflected in the generally higher quality of visualized LFPs [40].

#### EDS characterization

**Fig. 7:**
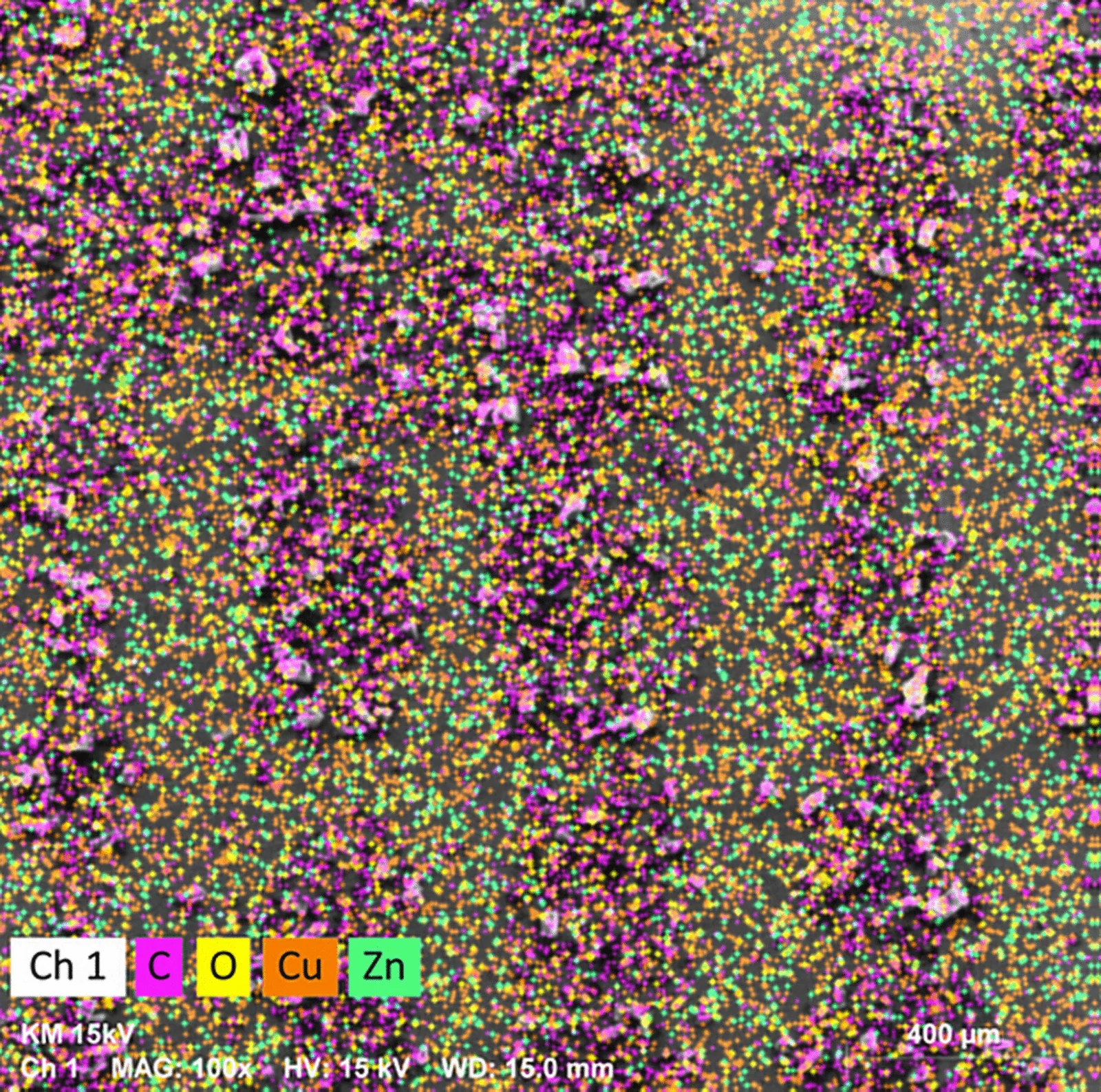
EDS spectrum of a fingerprint visualized with CUR on brass

The elemental composition of the model metallic materials (Fig. 7, S3–S5; see Supplementary Materials) on which the LFPs were visualized was confirmed using EDS analysis. This confirmed that areas corresponding to the papillary lines of the fingerprints exhibited the highest concentrations of carbon and oxygen (Fig. S6, see Supplementary Materials). These elements are indicative of organic compounds, including both sebaceous materials from the skin and CUR itself [22, 24]. The identification of carbon and oxygen in areas outside the fingerprint suggests that these elements are primarily introduced through the adsorption of organic dyes, rather than being inherent to the metallic substrate [41]. In addition to these elements, components of the underlying metal material were also identified. These findings correlate with previous studies [41] demonstrating the interaction between metal substrates and organic compounds during the LFP visualization process. The visualized markers are represented in pink (carbon) and yellow (oxygen), whereas the substrate shows predominantly orange and green colors, representing Cu and Zn (Fig. 7).

EDS provided a crucial insight into site-specific composition, showing that organic adsorbates, specifically fingerprint residues, are much more concentrated on the papillary lines compared to surrounding areas, thereby supporting the hypothesis that the dye effectively binds to the organic materials prevalent in the fingerprints [41].

#### Vibrational spectroscopic characterization

**Fig. 8:**
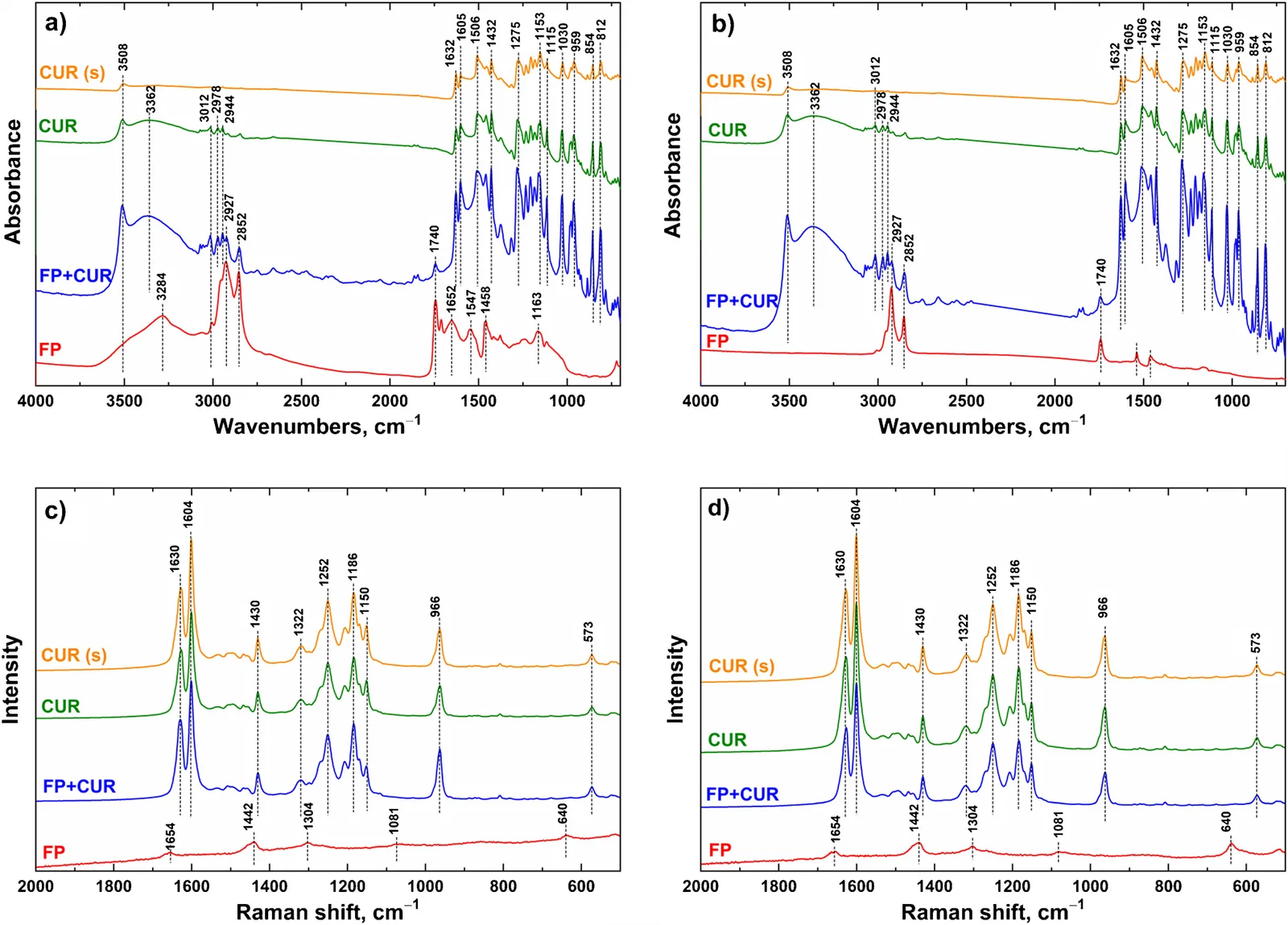
FTIR spectra obtained on **a** stainless steel and **b** brass and Raman spectra obtained on **c** stainless steel and **d** brass. In each spectrum, four reference measurements are shown: FP, sebaceous LFP; FP + CUR, fingerprints visualized using CUR; CUR, CUR deposited on the metal substrate; and CUR(s), solid CUR powder

When comparing spectra in Fig. 8, a striking difference is observed between the LFP spectra on steel and brass (Fig. 8a, b). In both spectra, the main bands of sebaceous components were detected at 1740 cm^–1^ (C = O stretching vibrations of saturated esters), 1458 and 1378 cm^–1^ (CH_3_ and CH_2_ symmetric bending vibrations), and 1163 cm^–1^ (CH_2_ twisting and rocking vibrations). The band with a maximum at 3009 cm^–1^ belongs to the = CH stretching vibrations of unsaturated fatty acids, glycerides, and wax esters of sebaceous components [42, 43]. In the case of steel, additional spectral features are observed, including a broad band with a maximum at 3284 cm^–1^ (hydrogen-bonded O–H stretching vibrations of human skin), the bands with maxima at 1652 and 1547 cm^–1^ (amide I and amide II of skin debris) [44]. In the case of brass, these bands are missing. These findings indicate that the interaction between the substrate (steel or brass) and the materials transferred from the papillary ridges of the fingers to the surface during contact differs. In the case of steel, both eccrine and sebaceous components are detected, whereas in the case of brass, the sebaceous components prevail. During adsorption in a water medium, the eccrine component dissolves and is removed from the surface. Differences in the visualization of LFP on Cu and stainless steel surfaces may be influenced by the relative contributions of eccrine and sebaceous components in the fingerprint residue. In particular, the improved visualization observed on steel surfaces can be attributed not only to the formation of non-covalent curcumin–lipid interactions, but also to interactions between curcumin and metal ions [16], which may affect its adsorption and photophysical properties.

The distinct spectral differences suggest divergent interactions between the materials from the papillary ridges of fingers and the substrate surfaces. On steel, the combination of eccrine and sebaceous components suggests retention of moisture and proteins, which may enhance fingerprint adhesion and persistence. Conversely, the predominance of sebaceous components on brass indicates that the eccrine component likely undergoes solubilization in water, resulting in its removal during the fingerprint deposition process. This observation aligns with previous findings that highlight the differing affinities for skin secretions to various materials [45].

After adsorption, CUR exhibits a new broad band with a maximum at 3362 cm^–1^ and enhancement of the intensities of the peaks situated at 1605 and 1458 cm^–1^, corresponding to the molecular vibrations of CUR’s structure associated with C = C and C = O bonds, respectively [46]. Strong adsorption occurs especially in the presence of the fingerprint. These results suggest that CUR is present in water medium in the form with one OH side group (instead of only two C = O groups) and adsorption is provided via this group.

The characteristic infrared bands of CUR are clearly observed in the spectrum, with their detailed assignments summarized in Table 2. The fingerprint region, including the distinct band at 1740 cm⁻^1^, is well resolved.

**Table 2:** FTIR band assignments of CUR

**Peak assignment**	***ν* (cm**^**−1**^**)**	**Ismail et al. **[47]	**Gunaskeran et al. **[48]
*ν*(OH)	3508	3508	3551
*ν*(C = C), *ν*(C = O)	1605	1628	1626, 1602
*ν*(C = O), *δ*(CCC), *δ*(CC = O)	1506	1512	1509
*ν*(COH), *δ*(CCC_Ar_), *δ*(CCH_Ar_)	1432	1427	1429
*δ*(CCC_Ar_), *δ*(CCH_Ar_), *δ*(COH)	1275	1277	1275
*δ*(CCH_Ar_)	1153		1142
*ν*(O–CH₃); *δ*(CCH_Ar_)	1115		1113
*δ*(CCH_Ar_), *δ*(CH₃)	1030		1025
*ν*(C = O); *δ*(COH)	959		962, 957
*δ*(CCH)	854		855
*δ*(CCH_Ar_)	812		821

Overall, the spectral data suggests a multifaceted interaction profile for CUR, heavily dependent on the structural context provided by substrate surfaces and hydration layers. The observed adsorption phenomenon is driven by the presence of hydroxyl groups, which facilitate strong molecular interactions via hydrogen bonding.

In contrast to the FTIR spectra (Fig. 8a, b), the Raman spectra (Fig. 8c, d) of pristine fingerprint residues on steel and brass are virtually identical. The spectra exhibit bands at 1654 cm⁻^1^ ν(C = O), 1442 cm⁻^1^ δ(CH₂), 1081 cm⁻^1^ ν(C–C), and 640 cm⁻^1^ δ(N–C = O) [42]. This indicates that Raman scattering is predominantly sensitive to various alkyl acetate components present in the fingerprint residues.

The Raman spectrum of CUR exhibits bands with maxima at 1630 cm⁻^1^ (C = O stretching vibrations), 1604 cm⁻^1^ *ν*(C = C), 1430 cm⁻^1^ *ν*(COH); *δ*(CCC_Ar_) and *δ*(CCH_Ar_), 1322 cm⁻^1^ *δ*(CCH) and *δ*(CCH_Ar_), 1252 cm⁻^1^ *δ*(CCH) and *δ*(COH), 1186 cm⁻^1^ *δ*(CH₃) and *δ*(COH), 1150 cm⁻^1^ *δ*(CCH_Ar_), 966 cm⁻^1^ *ν*(C = O), and 573 cm⁻^1^ [49]. These assignments are consistent with the functional group vibrations observed in the FTIR spectra, facilitating direct comparison of fingerprint residues with and without CUR treatment.

#### Effect of fingerprint aging

In practical forensic applications, LFPs are often recovered after a certain period rather than immediately after deposition. Since the aging of LFP residues may significantly influence visualization efficiency, an aging study was performed to evaluate the performance of CUR-based visualization on aged LFPs.

**Fig. 9:**
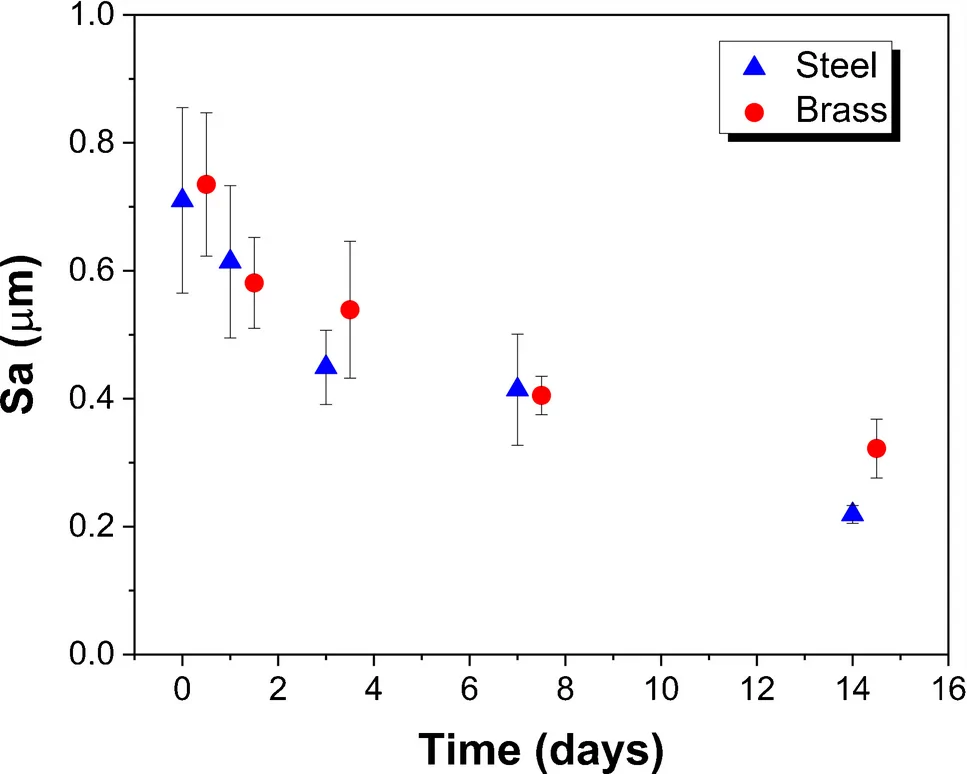
Evolution of the surface roughness parameter Sa of developed LFPs during aging on brass (red) and steel (blue)

On reactive metals, particularly brass, LFPs could be successfully visualized even 2 weeks after deposition. The Sa values gradually decreased from 0.735 µm for fresh LFPs to 0.322 µm after 2 weeks, corresponding to partial degradation of sebaceous residues and a reduction in topographical contrast between ridges and the background (Fig. 9; Table S3, see Supplementary Materials). Nevertheless, ridge details remained clearly distinguishable even at longer aging intervals.

On steel, a significantly faster deterioration of visualization quality was observed. While fresh LFPs provided high contrast and well-defined ridge detail, the visualization quality decreased markedly after 1 week. Although the fingerprints were still interpretable at this stage, the ridge detail became less distinct. After 2 weeks, the LFPs could no longer be reliably visualized. This trend corresponded well with the profilometric data, where the Sa value decreased from 0.710 µm for fresh LFPs to 0.219 µm after 2 weeks (Fig. 9; Table S4, see Supplementary Materials). The reduction in surface roughness was more pronounced than on brass and was associated with the loss of detectable ridge detail.

The observed decrease in Sa values with aging time can be attributed to the gradual drying, degradation, and redistribution of sebaceous components of the LFPs. Sebaceous LFPs consist predominantly of lipid components with low volatility; however, these compounds may progressively oxidize, diffuse across the surface, or be partially removed by environmental influences. The reduction in the amount of residue results in decreased selectivity in curcumin deposition, and consequently, lower contrast in the visualized ridge patterns.

The substantially higher stability of LFPs on brass compared to steel is likely related to differences in the chemical reactivity of the two metals toward LFP residues. This observation is consistent with the findings of Bond et al., who demonstrated that LFP residues can initiate localized corrosion processes on metallic surfaces, with copper-containing alloys showing a pronounced tendency to form stable corrosion patterns corresponding to LFP ridges. These processes may lead to the formation of a locally modified surface layer containing copper and zinc oxides that preserve ridge structures even after partial loss of organic residues [50]. On brass, a relatively stable template corresponding to the LFP ridges may therefore develop, supporting subsequent dye deposition even after prolonged aging.

In contrast, similarly stable surface modifications are unlikely to form on steel, as suggested by the general corrosion study of Graedel et al., and visualization is therefore primarily dependent on the presence of the original organic residues [51]. Once the amount of sebaceous material decreases below a critical level, curcumin deposition is no longer sufficiently selective, and the LFPs cannot be reliably visualized.

#### Quality of the visualized LFPs and assignment of minutiae on real objects

In dactyloscopic practice, second-level fingerprint features (minutiae) are routinely used for personal identification. To evaluate the practical applicability of dye-based LFP visualization, minutiae were assigned to selected real objects and assessed against the legal criteria applied in the CZ, which require a minimum of 10 distinct characteristic points for forensic usability. Except for LFPs contaminated with cosmetic products, all evaluated traces met or exceeded this minimum threshold.

**Fig. 10:**
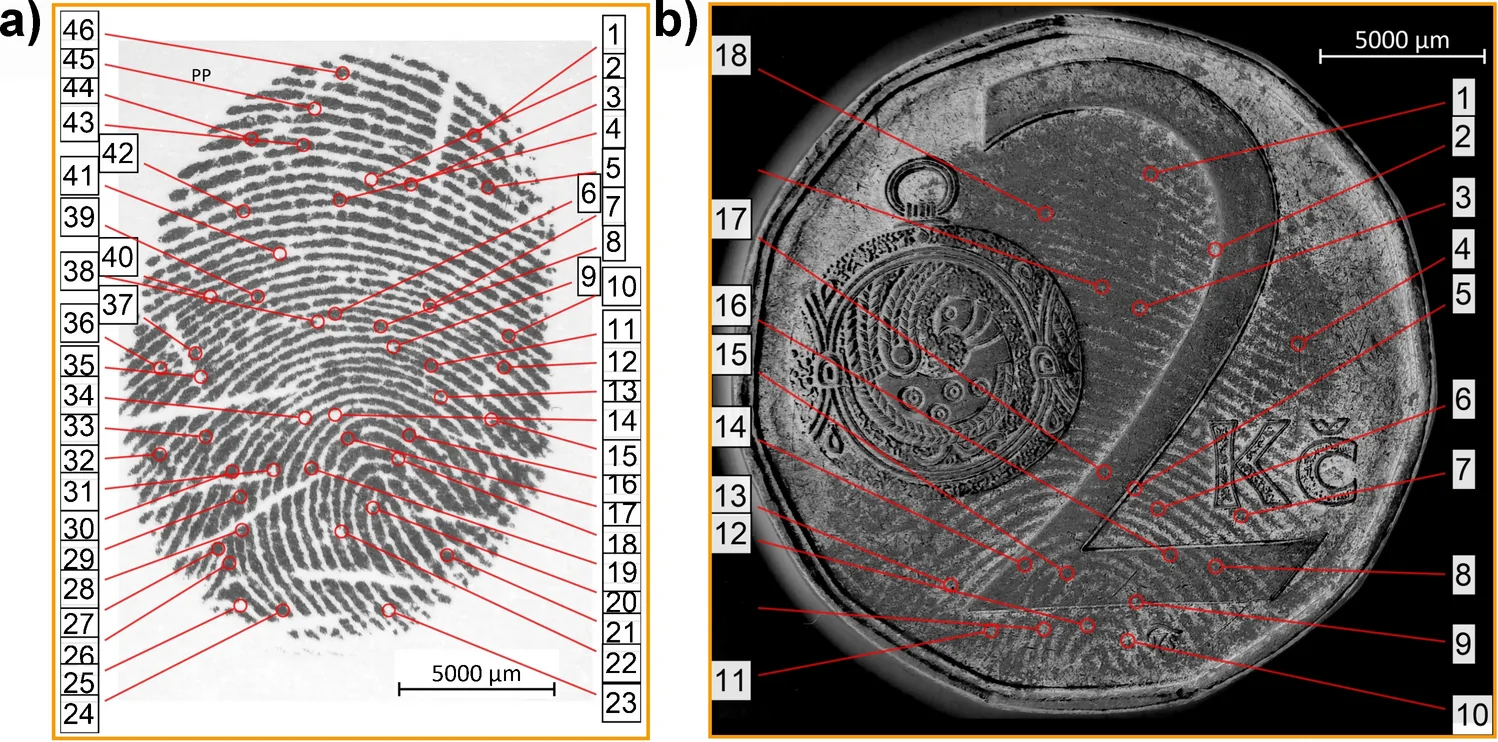
LFPs visualized with **a** fingerprint ink on paper and **b** CUR on a 2 CZK coin. Assigned minutiae are indicated in both images

As an illustrative example, the reference fingerprint printed on paper using PrintMatic Flawless printing ink contained 46 identifiable minutiae (Fig. 10a), of which 18 corresponding minutiae were successfully assigned on a 2 CZK coin (Fig. 10b), clearly exceeding the identification threshold.

Fingerprints obtained from knife blades (“Visualization of LFPs on metal substrates,” Fig. 4d) were further used to assess the quality of fingerprint visualization and papillary ridge detail. Trace no. 2 exhibited extensive smearing and poorly readable papillary ridges, resulting in an insufficient number of minutiae for individual identification. Trace no. 3 showed reduced ridge clarity with partial edge smearing. Although the number of minutiae was below the identification threshold, the trace remained suitable for exclusionary comparison. In contrast, trace no. 1 provided well-defined papillary ridges and a sufficient number of characteristic points to support individual identification despite partial central smearing. The highest-quality result was obtained for trace no. 4, which exhibited excellent ridge clarity across the entire contact area, with the number of identified minutiae far exceeding the minimum requirement and with fine details such as pores and wrinkles clearly discernible.

Overall, insufficient minutiae in non-identifiable traces are best attributed to pre-existing contamination or mechanical degradation of the papillary pattern rather than to limitations of this visualization approach.

## Conclusion

This work demonstrates that CUR is an effective, environmentally friendly agent for the visualization of LFPs on metal substrates. High-quality papillary ridge contrast and clear minutiae were achieved on several metals and real objects, particularly stainless steel, Ni-plated surfaces, and Al, as well as on model brass and Cu substrates under controlled surface conditions. In contrast, surface modification of real Cu- and brass-based objects resulted in non-selective background staining, reflecting altered surface chemistry and defining the current operational limits of the method.

The combination of optical imaging, profilometry, and SEM/EDS provided comprehensive insight into the morphology and molecular composition of LFPs developed using CUR. Spectroscopic analyses revealed that the visualization performance of CUR is driven by its selective interaction with organic LFP residues, particularly lipid-rich components, rather than by non-selective adsorption on the metal surface. Furthermore, aging experiments demonstrated that the long-term preservation of LFP papillary ridge structures is not governed solely by the drying and degradation of organic residues but is strongly influenced by interactions between LFP residues and the metallic substrate, with reactive metals exhibiting a substantially greater ability to preserve ridge patterns over extended periods. Overall, these results identify CUR as a robust green alternative for targeted LFP detection on selected metal substrates and underline its potential for further development within sustainable forensic and bioanalytical methodologies.

## Supplementary Information

Below is the link to the electronic supplementary material.

Supplementary file1 (DOCX 4.85 MB)

## Data Availability

Data will be made available on request.
