# Supplementary material for: Surface characterization of curcumin-visualized latent fingerprints on metal surfaces
Source: Anal Bioanal Chem. 2026 Mar 22;418(18):5979–92. doi: 10.1007/s00216-026-06445-x (PMC13541949; doi:10.1007/s00216-026-06445-x)
Supplement: Supplementary file 1 [file 216_2026_6445_MOESM1_ESM.docx]

# Supplementary Materials

**Surface Characterization of Curcumin-Visualized Latent Fingerprints on Metal Surfaces**

**Kristýna Havelková^a^, Jaroslav Otta^b^, Miroslava Trchová^c^, Tomáš Tobrman^d^, Petr Vrablic^e^, and Gabriela Broncová^a*^**

^a^ Department of Analytical Chemistry, Faculty of Chemical Engineering, University of Chemistry and Technology, Prague, Technicka 5, 166 28 Prague 6, Czech Republic

^b^ Department of Physics and Measurements, Faculty of Chemical Engineering, University of Chemistry and Technology, Prague, Technicka 5, 166 28, Prague 6, Czech Republic

^c^ Central Laboratories, University of Chemistry and Technology, Prague, Technicka 5, 166 28, Prague, Czech Republic

^d^ Department of Organic Chemistry, Faculty of Chemical Technology, University of Chemistry and Technology, Prague, Technicka 5, 166 28 Prague 6, Czech Republic

^e^ Criminalistics Institute Prague, Police of the Czech Republic, Bartolomejska 310/12, 110 00 Prague, Czech Republic

*e-mail: [Gabriela.Broncova@vscht.cz](mailto:Gabriela.Broncova@vscht.cz)

This file contains the supplementary materials about this article including figures and tables that are not shown in the main manuscript.

**Contents of the Supplementary Materials**

Table S1 Chemical composition of metallic substrates (related to *Preparation of substrates and LFP deposition* of the main text)

**Fig. S1** XRF spectra of selected metallic objects: **a)** brass, **b)** steel, **c)** Cu, **d)** Al, **e)** key, **f)** 1 CZK coin, **g)** 10 CZK coin, **h)** 20 CZK coin, and **i)** knife (related to *Preparation of substrates and LFP deposition* of the main text)

**Fig. S2** Visualization of real metal objects with sebaceous latent fingerprints (LFPs) using curcumin (CUR) in **a)** a Petri dish and **b)** a test tube (related to *Visualization procedure* of the main text)

**Table S2** Deposition time of developed LFPs on model and real metal objects (related to *Visualization of LFPs on metal substrates* of the main text)

**Fig. S3–S5** EDS spectra of LFPs visualized with CUR on different metallic substrates: **Figure S2**, aluminum; **Figure S3**, copper; **Figure S4**, steel (related to *EDS characterization* of the main manuscript)

**Fig. S6** Qualitative EDS analysis of carbon and oxygen in surface residues after sebaceous LFPs visualization with CUR (related to *EDS characterization* of the main manuscript)

**Table S3** Aging of visualized fingermarks on brass documented by stereoscopic microscopy and surface profilometry (related to *Effect of fingerprint aging* of the main manuscript)

**Table S4** Aging of visualized fingermarks on steel documented by stereoscopic microscopy and surface profilometry (related to *Effect of fingerprint aging* of the main manuscript)

**ED-XRF Measurement Conditions**

**ED-XRF.** The elemental composition of the metallic substrates was determined using a portable energy-dispersive X-ray fluorescence (XRF) spectrometer ElvaX Mobile (RMI, CZ). The instrument is equipped with an X-ray tube with a rhodium anode and a large-area silicon drift detector (SDD) with an energy resolution of 135 eV at 20,000 counts per second and a maximum count rate of 200,000 counts per second. The detector is thermoelectrically cooled to −40 °C. The spectrometer includes a motorized four-position filter carousel enabling automatic exchange of primary beam filters (Ti 400 µm, Al 800 µm, Ni 100 µm, Ni 300 µm + Al 300 µm, and measurements without a filter). The high-voltage generator operates in the range of 4–50 kV (100 V step) with an adjustable tube current of 0–100 µA (0.2 µA step), with a maximum equivalent tube power of 50 W. Measurements were performed under ambient laboratory conditions. The excitation parameters were optimized according to the analyzed material, and quantitative evaluation was carried out using the manufacturer’s software based on the fundamental parameters (FP) method.

Table S1 Chemical composition of metallic substrates

| **Substrate** | **Material Type** | **Elemental Composition** | **Data Source** |
| --- | --- | --- | --- |
| **Brass** | Alloy | Cu (63.9 wt.%); Zn (36.1 wt.%); Fe, LE (<0.1 wt.%) | XRF |
| **Steel** | Alloy | Fe (71.4 wt.%); Cr (18.3 wt.%); Ni (7.9 wt.%); minor Mn, P, Si, Al, V, Cu, Mo, LE (<1.5 wt.% each) | XRF |
| **Copper** | Pure metal | Cu (99.8 wt.%); minor Sb, Zn, Fe, LE (<0.1 wt.% each) | XRF |
| **Aluminum** | Pure metal | Al (88.4 wt.%); LE (7.3 wt.%); minor Cl, P, Fe, Ca, S, V, Zn, Ga, Mn, LE (<3 wt.% each) | XRF |
| **Key** | Alloy | Ni (56.8 wt.%); Fe (40.9 wt.%); minor I, P, Mn, Al, Pb, Mo, LE (<1.5 wt.% each) | XRF |
| **2 and 5 CZK** | Ni-plated steel | Steel core plated with Ni  Ni (89.9 wt.%); Fe (4.4 wt.%); minor Cl, I, K, LE (<3 wt.% each) | CNB [1]  XRF |
| **10 CZK** | Copper-plated steel | Steel core clad and galvanically coated with Cu  Cu (99.8 wt.%); minor Zn, Fe, LE (<0.1 wt.%) | CNB [1]  XRF |
| **20 CZK** | Brass-plated steel | Steel core clad with Cu–Zn alloy (Cu 75 wt.%; Zn 25 wt.%); and electroplated with Cu–Zn alloy (Cu 72 wt.%; Zn 28 wt.%)  Cu (75.7 wt.%); Zn (24.2 wt.%); minor Fe, LE (<0.1 wt.%) | CNB [1]  XRF |
| **Knife** | Alloy | Fe (84.9 wt.%); Cr (13.6 wt.%); minor P, Mn, Si, Al, Ni, V, S, LE (<0.5 wt.% each) | XRF |

*Abbreviations: LE – Light elements (elements with low atomic numbers, up to Na, according to the instrument software classification).*

*Kα (Ka in the graph) – the most intense characteristic X-ray line of an element observed in XRF spectra, corresponding to the transition of an electron from the L shell to the K shell.*

Fig. S1 XRF spectra of selected metallic objects: a) brass, b) steel, c) Cu, d) Al, e) key, f) 1 CZK coin, g) 10 CZK coin, h) 20 CZK coin, and i) knife

**Fig. S2** Visualization of real metal objects with sebaceous LFPs using CUR in **a)** a Petri dish and **b)** a test tube

Table S2 Deposition time of developed LFP on model and real metal objects

| **Model Metal** | **Time [min]** | **Real Object** | **Time [min]** |
| --- | --- | --- | --- |
| Brass | 10 | Key | 30 |
| Steel | 30 | 2 and 5 CZK | 60 |
| Copper | 10 | 10 CZK | 30 |
| Aluminum | 10 | 20 CZK | 30 |
|  |  | Knife | 60 |

Fig. S3 EDS spectrum of a LFP visualized with CUR on aluminum

Fig. S4 EDS spectrum of a LFP visualized with CUR on copper

Fig. S5 EDS spectrum of a LFP visualized with CUR on steel

The EDS analysis confirms the presence of carbon and oxygen on all metallic substrates investigated after LFPs visualization with CUR. Carbon is associated with organic components originating from both sebaceous LFPs residues and the CUR. Oxygen is also related to organic material. However, its signal may additionally arise from native oxide layers present on metallic substrates, particularly in the case of aluminum, copper, brass, and steel. These results are mainly related to the limited surface sensitivity of EDS and the possible contribution from underlying substrates.

EDS elemental composition of residues on metal surfaces

Atomic %

| Materials | C (%) | O (%) |
| --- | --- | --- |
| Steel | 63.28 | 13.56 |
| Aluminum | 53.88 | 9.52 |
| Brass | 55.58 | 9.36 |
| Copper | 56.6 | 9.81 |

Fig. S6 Qualitative EDS analysis of carbon and oxygen in surface residues after sebaceous LFPs visualization with CUR

**Table S3** Aging of visualized fingermarks on brass documented by stereoscopic microscopy and surface profilometry

| **Aging time (days)** | **Visualized fingerprint (left) and the corresponding profilometric image (right)** |
| --- | --- |
| 0 |  |
| 1 |  |
| 3 |  |
| 7 |  |
| 14 |  |

**Table S4** Aging of visualized fingermarks on steel documented by stereoscopic microscopy and surface profilometry

| **Aging time (days)** | **Visualized fingerprint (left) and the corresponding profilometric image (right)** |
| --- | --- |
| 0 |  |
| 1 |  |
| 3 |  |
| 7 |  |
| 14 |  |

**References**

[1]	Czech National Bank, Czech coins, https://www.cnb.cz/cs/bankovky-a-mince/mince/ (accessed February 23, 2026).
